# Hyperbolic Modeling of Subthalamic Nucleus Cells to Investigate the Effect of Dopamine Depletion

**DOI:** 10.1155/2017/5472752

**Published:** 2017-09-06

**Authors:** Mohammad Daneshzand, Miad Faezipour, Buket D. Barkana

**Affiliations:** ^1^Department of Computer Science and Engineering, University of Bridgeport, Bridgeport, CT, USA; ^2^Department of Electrical Engineering, University of Bridgeport, Bridgeport, CT, USA

## Abstract

To investigate how different types of neurons can produce well-known spiking patterns, a new computationally efficient model is proposed in this paper. This model can help realize the neuronal interconnection issues. The model can demonstrate various neuronal behaviors observed in vivo through simple parameter modification. The behaviors include tonic and phasic spiking, tonic and phasic bursting, class 1 and class 2 excitability, rebound spike, rebound burst, subthreshold oscillation, and accommodated spiking along with inhibition neuron responses. Here, we investigate the neuronal spiking patterns in Parkinson's disease through our proposed model. Abnormal pattern of subthalamic nucleus in Parkinson's disease can be studied through variations in the shape and frequency of firing patterns. Our proposed model introduces mathematical equations, where these patterns can be derived and clearly differentiated from one another. The irregular and arrhythmic behaviors of subthalamic nucleus firing pattern under normal conditions can easily be transformed to those caused by Parkinson's disease through simple parameter modifications in the proposed model. This model can explicitly show the change of neuronal activity patterns in Parkinson's disease, which may eventually lead to effective treatment with deep brain stimulation devices.

## 1. Introduction

Precise explanation of a group of coupled spiking neurons is far beyond our understanding, but if we go deeper and consider a single neuron alone, we can see that any spiking pattern depends on the presence of the ion channel in the cell membrane. What happens that actually leads to different types of neuronal spiking is the existence of various ionic currents [1]. These spiking patterns can be classified into four major groups: regular spiking, fast spiking, intrinsic bursting, and continuous bursting neurons [2]. Several models that try to investigate cell spiking characteristics have been introduced in the literature. These models can be categorized into two main groups. The first group are biologically inspired models that aim to take into account the ionic variation through cell membrane and study the effect of different ions like calcium and potassium on the membrane potential [3–6]. The second group of brain cell modeling research efforts use pure mathematical models that might not consider a lot of biological features, but at the same time they are able to generate different neuronal responses [7–10].

Definition of a practical model that can address real brain problems through a wide variety of disorders such as sleep apnea, seizure, and attention deficiency is to consider biological features of a cell along with less complex mathematical equations. Since any brain disorder involves a group of neurons, a computationally efficient neuron model that can help understand the behavior of a large group of coupled neurons, while providing low computational complexity, is of high significance [11].

One significant neuronal response is called burst firing, which can be seen in different cells of the brain such as hippocampus, cortical neurons, mid-brain, and subthalamic nucleus. Interestingly, this bursting behavior can be observed in dopaminergic (DA) neurons in ventral mid-brain which are related to Parkinson's disease when there is a loss in the amount of these DA and substantia nigra cells [12, 13].

**Figure 1:**
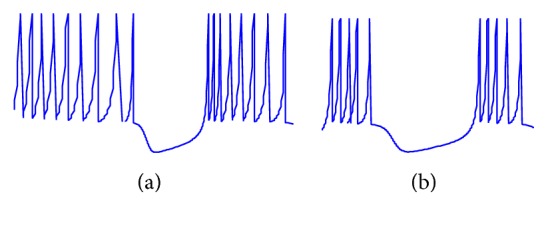
Schematic representation of action potential in STN cells in absence of dopamine (a) and in presence of dopamine (b).

Substantia nigra is considered as a nucleus part in Parkinson's disease with a crucial role in the motor center of the brain. Subthalamic neurons also generate complex patterns in which dopaminergic innervation will change synaptic transition and ion channels and, furthermore, will affect the firing patterns [14]. In Parkinson's disease, depletion of dopamine modifies synaptic transition, causing abnormal firing patterns in subthalamic nucleus. It has been observed in humans with Parkinson's disease that the subthalamic nucleus would show bursting patterns more than normal cases along with less single spiking patterns (see Figure 1) [15]. In this paper, the bursting pattern of subthalamic nucleus in Parkinson's disease will be studied through a mathematical relationship, capable of modeling this kind of abnormality.

The rest of this paper is organized as follows. In Section 2, we propose a new mathematical model for spiking responses generated by brain cell, using an extension over the Izhikevich model. We will show how our model can generate all the well-known spiking patterns while providing more feasibility over previous methods. In Section 3, different spiking patterns of subthalamic nucleus neurons are presented and investigated through the proposed model. Effect of dopamine on subthalamic nucleus neurons and its relation with Parkinson's disease are elaborated in Section 4. This section also presents the results of derived spiking patterns in presence and absence of dopamine using our model. The paper ends with a concluding discussion in Section 5.

## 2. Proposed Model

In this section, we propose a mathematical model to describe the firing behavior of substantia nigra cells in order to investigate single neuron response in healthy subjects and those with Parkinson's disease.

### 2.1. Hyperbolic Model of Neurons

The hyperbolic model can be used to study the neuronal firing patterns of cells with dopamine deficiency. This model is an extension over previous bidimensional models such as the Izhikevich model [9] and Adaptive Exponential Integrate-and-Fire (AdEx) model [10] which allows more flexibility by simply reducing the number of parameters in order to generate various spiking patterns. Due to the ambiguous nature of neuronal responses and the partial differential equations that can model them, we consider a hyperbolic differential equation to model all types of bursting behavior. This idea comes from the vast applications of hyperbolic functions to solve differential equations. The proposed mathematical model can be formulated as follows:

(1) dvdt=αcosh⁡v−vrestβ−1−ϵ−H+I

(2) dHdt=τHnHv−H.

Equation (1) implies the fact that the membrane potential of a cell can be considered as a differential equation. Although many models are designed based on biological phenomena in cells, here, we aim to generate a mathematical model focusing on the computational cost. In this equation, we considered the insulation of cell membrane around a neuron as a capacitor which is defined by parameter *α*. Current *I* in this model represents the ionic movement through cell gates (inward calcium and sodium ionic velocity), resulting in action potential or voltage spikes. In existence of current *I*, the membrane voltage would increase and generate spiking patterns until it reaches a threshold value and should be reset to a resting state via *v*_rest_. The sharpness of spikes can be modified through *β*. The parameter *ϵ* is an experimentally determined parameter, related to the general gate voltage of inward calcium and sodium and outward potassium currents.

Equation (2) can determine the rate of spikes along with their resting and peak times. Simply, by changing parameters in these equations, one can observe different types of neuronal firing. As shown in Figure 1, with the selection of *α* = 75000, *β* = 1000,   *v*_rest_ = −62.5, *ϵ* = 16,   *τ*_*H*_ = 0.02, and  *n*_*H*_ = 0.2, we can see the tonic bursting behavior of neurons spiking. Note that any time the membrane potential reaches its peak, this voltage will be reset to the resting state, and a delay time to *H* called *H*_Delay_ should be added. A simple MATLAB version of this model has been added to enselab.med.yale.edu/modeldb. By changing the parameters, one can generate various firing patterns.

### 2.2. Advantages of Hyperbolic Model

The similarities between the hyperbolic model and the Izhikevich model or AdEx model are as follows: same bifurcation patterns in all models, ability to generate various firing patterns, and easy and fast simulation and implementation time. The proposed hyperbolic model has advantages over the Izhikevich model and AdEx model in terms of computation cost due to the hyperbolic membrane voltage in comparison with quadratic and exponential functions, as seen in Table 1. We tested the simulation time to generate 10 different spiking patterns and averaged overall. The simulation time for the proposed hyperbolic model was the lowest, which is crucial in modeling neural population. The slow upswing action potential in the Izhikevich model and AdEx model is improved by the hyperbolic function (Table 1). The hyperbolic model was also able to linearize the subthreshold voltage similar to realistic recordings of STN cells. Based on the recordings of STN firing patterns [17], a combination of linear and exponential models can achieve better results in which the nonlinearity of hyperbolic functions can obtain this goal. Finally, our proposed hyperbolic model provides better quantitative fits to voltage traces [18]. We tested a single tonic spike generated in our model (*S*_1_) with the actual recordings of STN neurons (*S*_2_) using the Cross Correlation (CC) measure as shown in the following equation:

(3) CCS1,S2=∑t=1LS1−meanS1S2−meanS2∑t=1LS1−meanS12∑t=1LS2−meanS22.

*L* is the length of a spike, and, as shown in Table 1, the hyperbolic model and AdEx model obtained the maximum CC values with actual recording of STN spikes [16].

Furthermore, the hyperbolic model is able to generate switching firing patterns of STN neurons caused by Parkinson's disease. This switching behavior from tonic to burst cannot be obtained by Izhikevich model or AdEx model. Physiologically inspired models can obtain slightly higher CC [19] but the large parameter set of these models will significantly reduce the computational performance in terms of simulation and upswing time (Table 1).

In terms of number of parameters, the hyperbolic model has 5 parameters in total (excluding the reset potential). The number of parameters for Izhikevich model and AdEx model is 4 and 6, respectively. However, the parameters *α* and *β* in our model can be set to a fixed value (75000 and 1000, resp.) and still be able to generate various neuronal patterns. The reason that *α* and *β* values are slightly different in Table 2 is to obtain more clear firing patterns. Therefore, the main parameterization task in the proposed model is done with only 3 parameters (*ϵ*, *τ*_*H*_, and *n*_*H*_). This will significantly reduce the difficulty of the parameterization task.

**Figure 2:**
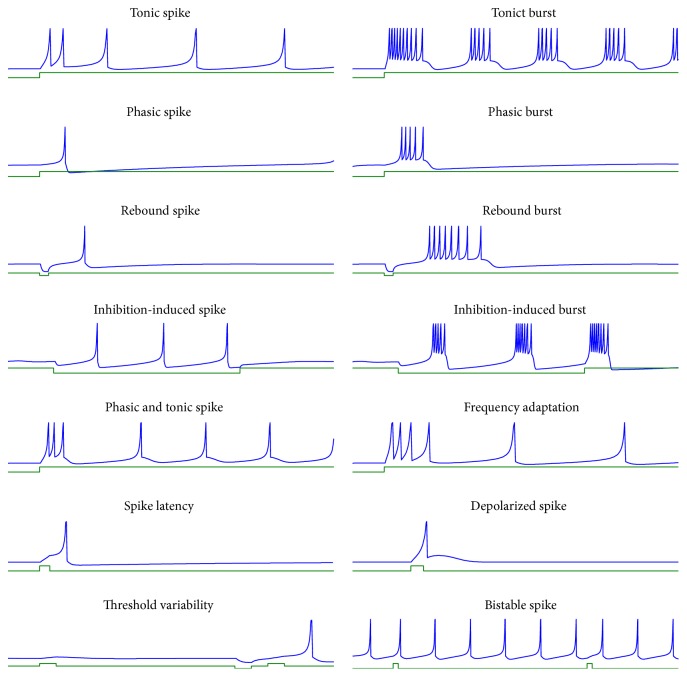
Different spiking patterns generated by our model. Green lines are the input currents to our model and blue plots are the output firing patterns.

Figure 2 shows various types of single neuronal responses that can be generated by this model. The responses include* tonic spikes* that happen within excitatory neurons under presence of stimuli. The* tonic burst *behavior mostly happens in neurons related to gamma frequency oscillations in the brain [20], in which, at any peak, a bursting behavior is seen before the spike goes to the resting state.* Phasic spike *and* phasic burst* behaviors happen when there is a constant stimuli neuron response only once and can show bursting behavior while spiking.* Rebound spike *and* rebound burst* responses are based on the anodal break excitation in excitatory membrane, which are mostly observed in thalamocortical neurons. The* inhibition-induced spike *and* burst* are also known as a behavior of the thalamocortical cells. When this behavior is combined with bursting rhythms, it is considered as a response of neurons while sleeping.* Phasic *and* tonic spikes *are conditions due to the situation when a stimulus neuron starts bursting responses. After a while, this behavior would be changed to tonic spikes. The* frequency adaptation *behavior is mainly known as the reduction in the frequency of spikes which happens mostly in the neocortex cells. The s*pike latency* behavior is sometimes based on the intensity of a stimulus, where a neuron will fire with a delay. Sometimes, after a spike, the membrane potential will be depolarized, which refers to the* depolarized spike* behavior. The threshold value of a neuron is not always fixed and depends on its previous spikes along with previous stimuli. Neuron spiking after consecutive small stimulus is a condition when the spiking threshold is changed and is called* threshold variability*. If a neuron response changes between resting and tonic or bursting spike states, this behavior is considered as* bistable spikes*. The parameter settings along with input currents for all the patterns in Figure 2 are shown in Appendix A. Furthermore, the dynamical analysis of the proposed hyperbolic model is discussed in Appendix B.

## 3. Subthalamic Nucleus Neurons Firing Patterns

**Figure 3:**
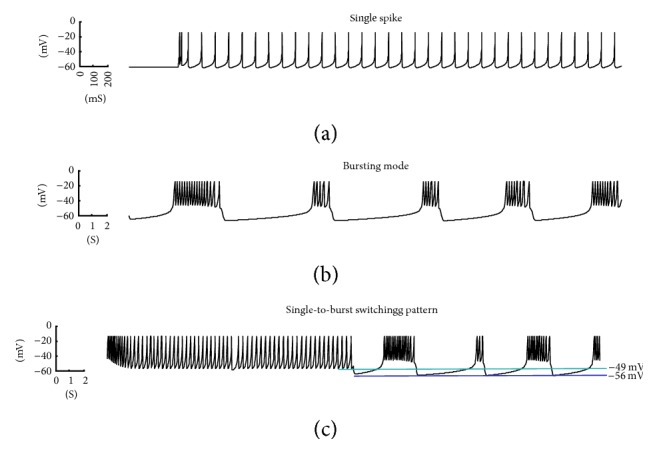
Single, burst, and switching mode of STN neurons spiking generated by our model. The amplitude and frequency of these spikes are within the range of recorded firing responses [16].

Subthalamic nucleus (STN) neurons show three different types of firing patterns: bursting, irregular, and rhythmic. The bursting patterns usually occur with high frequency spikes, which in humans would vary from 18 to 28 spikes per seconds [21]. Irregular patterns are considered as randomly rapid interspike firings. Finally, the rhythmic patterns generate single spikes with multiple peaks. It is shown by the measurement of STN spiking that both tonic and burst patterns exist [16]. Based on the current value of membrane potential, STN neurons can change their firing patterns from single-spike mode to bursting patterns. This transition between two modes is observed via our mathematical model. Recordings of STN neurons spikes reveal that the single spike's peak is between −35 and −70 mV and burst spikes generate the membrane potential of −42 to −60 mV [16].  Figure 3 shows single spiking, burst spiking, and the switching pattern from single spikes to burst spikes generated by our model using MATLAB. Table 2 shows the parameters setting for obtaining these STN patterns from our proposed model. The quantitative parameters of the results satisfy the actual recordings of STN spiking patterns provided by [16], as shown in Table 3.

**Figure 4:**
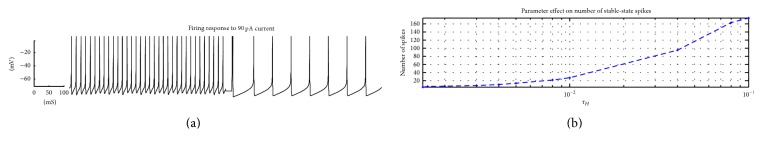
(a) Spike response of a STN neuron to constant DC current results in both high-frequency and stable-state mode. (b) Relation of *τ*_*H*_ to the number of spikes at the stable mode of STN neurons.

The average rate of STN neurons spikes is an important factor in the pathophysiology of Parkinson's disease [22]. Studies show that STN neurons can generate around 500 spikes per second while stimulated by an input current stimulus of 100–200 pA for 100–1000 ms duration [23]. Under constant current, these neurons spike with high frequency until they reach a maximum frequency and after that they have a resting state until they start spiking in a stable mode.  Figure 4 shows the response pattern of a STN neuron to constant current stimuli of 90 pA, resulting from our model. After a high-frequency state, we can see a stable pattern in which the number of spikes is directly proportional to *τ*_*H*_ in (2). The more we increase *τ*_*H*_, the number of spikes in the stable state would increase, which can be seen in Figure 4(b). The parameters for obtaining this pattern are also provided in Table 2.

## 4. STN Neurons Spiking Patterns due to Dopamine Depletion and Parkinson's Disease

STN neurons are under influence of dopamine through their receptors and signaling pathways. They will be depolarized under activation of postsynaptic dopamine receptors, causing high-frequency spikes. Dopamine depletion in STN neurons can cause several disorders in body movement. As of interest in this research investigation, loss of dopamine innervation is a significant symptom of Parkinson's disease.

**Figure 5:**
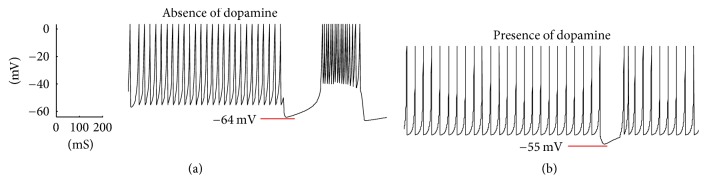
Absence (a) and presence (b) of dopamine in STN neuronal firing. In the presence of dopamine, rebound burst would decrease.

Scientists believe that there is a relation between excessive bursting patterns in STN neurons and Parkinson's disease [24]. The important question now is how the firing patterns of STN neurons, considering their apparent features, are related to Parkinson's disease. Generally, Parkinson's disease is accompanied by loss of dopamine innervation, leading to abnormal burst firing patterns in STN neurons. Why these bursting patterns in STN neurons diminish under existence of dopamine is still under debate among scientists [25, 26]. Researchers explained that dopamine may suppress synoptically triggered burst firing patterns; in the meantime, this suppression of burst firing patterns could be a result of a synaptic current. In Figure 5, we modified our model to obtain both firing patterns of STN neurons in presence and absence of dopamine, which has been recorded in related works [7]. The absence or presence of dopamine in our model is defined by parameters *τ*_*H*_ and *n*_*H*_ that represent the decay rate and sensitivity of spikes, respectively. *τ*_*H*_ is set to 0.02 and 0.1 to model the absence and presence of dopamine in STN neurons, respectively. *τ*_*H*_ is much lower in the case of dopamine depletion compared to the case when dopamine is present, which provides a faster decay time. Also, *n*_*H*_ is set lower for absence of dopamine in comparison with the presence of dopamine condition. This *n*_*H*_ parameter modification in case of dopamine depletion along with faster decay time due to low value of *τ*_*H*_ guarantees enough time for the transition of tonic spiking into burst firing patterns. In the presence of dopamine, the transition is almost diminished, providing a more tonic pattern (Figure 5) due to higher values of *τ*_*H*_ and *n*_*H*_.

**Figure 6:**
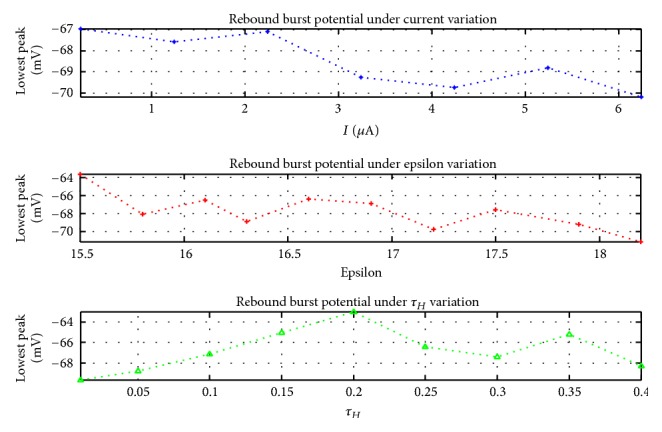
Rebound burst potential relation with parameters of our model. Vertical axis shows the rebound burst potential.

As shown in Figure 5, bursting patterns would diminish under presence of dopamine. Note that there is a significant decrease in rebound burst pattern in the presence of dopamine (red lines). Parameter values of our model for generating the responses in Figure 5 are shown in Table 2. The important feature here is how dopamine would improve the rebound burst in STN neurons. We discuss how changing parameters in our model can lead to different rebound burst potentials. The effect of these parameters can be seen in Figure 6, in which the value of rebound burst potential is calculated under variable parameters.

For *ϵ* values greater than 18.32, the firing pattern would change to tonic spike, meaning that this is the upper bound for *ϵ*. For *τ*_*H*_ larger than 0.41, the rebound burst would change to depolarized spike, which shows the upper bound for *τ*_*H*_. Finally, for *n*_*H*_, the lowest value in which the rebound burst appears is 0.24, and the maximum value to have a rebound burst pattern is 0.26.  Table 4 shows these parameters ranges and their resulting patterns.

Note that, in this table, in order to see the effect of each parameter in generating a specific pattern, the other two parameters should stay in the indicated range as shown in the 3rd and 4th rows.

## 5. Discussion

This work presented a mathematical model to generate various neuronal spiking patterns. The key advantage of this model is the fact that it has low computational complexity and can produce various neuronal firing patterns by only adjusting very few parameters. In other words, less parameters are involved in the proposed model to produce several patterns when compared to other works due to the inherent properties of hyperbolic functions.

**Figure 7:**
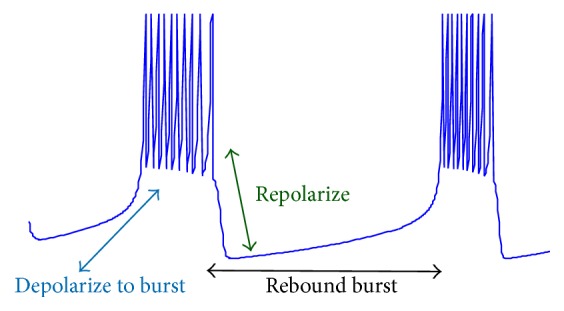
Different steps of burst firing mode.

Studies show that half of STN neurons might change their firing patterns from single spikes to bursting mode [16]. Due to impact of STN neurons on Parkinson's disease, we proposed a model to reflect these single-to-burst switching patterns. We showed how variation of parameters in our model leads to different types of spikes, which furthermore can be related to actual understanding of STN cells. As [16] stated, while in bursting mode, depolarization of membrane voltage occurs due to calcium entrance through calcium cannels of STN cells. This is shown in Figure 7 generated by our model.

Based on this biological fact, we can interpret our model and state that the depolarization in burst firing mode can be explained through the parameter *τ*_*H*_ of our model. The depolarization to burst phase is caused by low calcium current which can be generated in our model by setting *I* with a low value. After that, we have a bursting behavior modeled by parameters set shown in Table 2. Each spike in this state will slightly increase the calcium current and the increase in calcium velocity will activate potassium current [27]. In the next phase, the membrane potential would be repolarized and we can model this by adjusting the parameter *n*_*H*_. The burst spiking pattern would be repeated by a depolarization phase due to calcium shortage.

Understanding how this model can be related to physiological phenomena in STN cells plays a significant role in realizing how Parkinson's disease or dopamine deficiency would affect the STN neurons. In addition, it can be used to develop a platform for simulating a group of these STN neurons. This will also further help us figure out how we can change parameters, aiming at changing the spiking patterns, as declared in Table 4. As an example, increase of current *I* in (1) would decrease the value of the rebound burst or it will generate a bigger rebound burst that happens in dopamine deficiency (Figure 6). This current increase can be related to the actual velocity of calcium [28] and therefore we can state that calcium concentration in STN cells has an effect on Parkinson's disease or on dopamine deficiency disorders.

As a future direction, we aim to use the hyperbolic model for studying the neuronal firing patterns of other cells within the basal ganglia, which is directly related to Parkinson's disease [29]. Modeling various brain cells in the basal ganglia with hyperbolic equations and defining the connectivity between them can help us study the effect of Parkinson's disease on the basal ganglia. We can then implement this hyperbolic basal ganglia model on Field Programmable Gate Array (FPGA) digital hardware boards, which gives us the opportunity to study large-scale network of neural population. FPGA implementation also provides a platform to investigate the effect of neurophysiological mechanisms such as voltage-gated channels and synaptic activities on the behavior of neuronal network in a more realistic simulation time [30, 31].

## Figures and Tables

**Figure 8:**
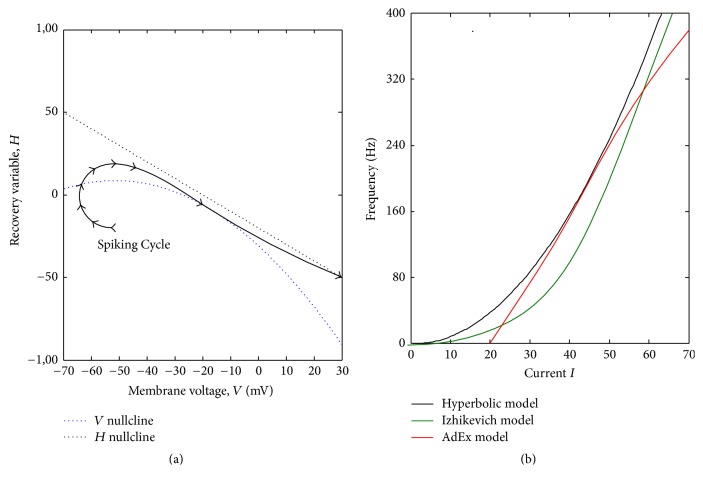
The phase portrait of tonic spiking pattern from the hyperbolic model (a) and the *F*-*I* curve of tonic spiking pattern (b).

**Table 1:** Comparison of hyperbolic model and well-known neuron models.

	Izhikevich	AdEx (Gerstner and Brette)	Humphries et al.	Hyperbolic
Number of parameters	4	6	9	3 to 5
Simulation time (s)	0.108	0.152	0.424	0.084
Upswing time (s)	0.0014	0.0011	0.0034	0.0005
CC	0.785	0.809	0.813	0.827

**Table 2:** Parameters setting for generated spikes according to (1) and (2).

Spiking patterns	Parameters					
	*α*	*β*	*v*_rest_	*ϵ*	*τ*_*H*_	*n*_*H*_
Single spike	75000	950	−62.5	16	0.021	0.2
Bursting spike	69000	1000	−61.5	16.1	−0.026	−1
Single to burst switching	71000	965	−62.5	15.7	−0.02	−1
Constant current	74000	1000	−55.5	16.6	0.1	0.25
Absence of dopamine	75000	1000	−62	16.2	0.02	0.2
Presence of dopamine	75000	1000	−62.5	16.9	0.1	0.36

**Table 3:** Spike characteristic results of our model in comparison with actual recordings of STN neurons.

	Single-spike mode		Bursting mode		Constant current 90 pA			
	Our model	Beurrier et al.	Our model	Beurrier et al.	Our model for high-frequency state	Wilson et al.	Our model for stable state	Wilson et al.
Spike amplitude (mV)	70.5	74.7	73.6	79.8	82.6	83.5	83.8	80.1
Duration of a spike (mS)	1.5	1.3	198	190	20	20	0.1	100
Spike max. peak (mV)	9.3	15.4	10.9	17.7	12.9	19.9	11.8	11.6
Spike min. peak (mV)	−62.8	−60.4	−62.1	−61.8	−69.9	−65.2	−72.8	−70.3

**Table 4:** Parameter range for different spiking patterns.

15.2 < *ϵ* < 18.32	*ϵ* > 18.32	0.02 < *τ*_*H*_ < 0.41	*τ*_*H*_ > 0.41	0.24 < *n*_*H*_ < 0.26	*n*_*H*_ > 0.26

Rebound burst	Tonic spike	Rebound burst	Depolarized spike	Rebound burst	Bistable spikes

−0.03 < *τ*_*H*_ < 0.12		15.4 < *ϵ* < 17.1		15.5 < *ϵ* < 16.95	

−1 < *n*_*H*_ < 0.3		−1 < *n*_*H*_ < 0.3		0.02 < *τ*_*H*_ < 0.34	

**Table 5:** Exact parameter set to generate spiking patterns of Figure 2 based on the proposed hyperbolic model.

	*α*	*β*	*ϵ*	*τ*_*H*_	*n*_*H*_	*I* (*μ*A)
Tonic spike	75000	950	16	0.021	0.2	Constant at 14
Tonic burst	75000	1000	16	0.02	0.24	Constant at 15
Phasic spike	75000	1000	16	0.02	0.25	Constant at 0.5
Phasic burst	75000	980	16.6	0.02	0.26	Constant at 1
Rebound spike	75000	1000	16	0.03	0.25	Single pulse with peak of −15
Rebound burst	74000	960	15	0.03	0.2	Single pulse with peak of −15
Inhibition-induced spike	75000	1000	16	−0.02	−1	Pulse with high peak at 80 and low peak at 75
Inhibition-induced burst	71000	1000	15.7	−0.026	−0.8	Pulse with high peak at 80 and low peak at 72
Phasic and tonic spike	73000	920	16.2	0.02	0.2	Constant at 10
Frequency adaptation	75000	1000	16	0.01	0.2	Constant at 30
Spike latency	74000	1000	16.1	0.02	0.18	Single pulse with peak of 7
Depolarized spike	75000	9500	16	1	0.2	Single pulse with peak of 20
Threshold variability	75000	1000	15.3	0.032	0.25	Consecutive pulses with peak at 1
Bistable spike	75000	950	16	0.1	0.26	Consecutive pulses with peak at 1.24

## Appendix

### A. Model Parameterization

Based on (1) and (2), there are 5 parameters (*α*, *β*, *ϵ*, *τ*_*H*_, and *n*_*H*_) along with a resting potential (*v*_rest_) and input current *I* which are needed to be determined in order to generate various spiking patterns. The relative parameter value to each spiking pattern is shown in Table 5. Note that *v*_rest_ is −62.5 mV for all spiking patterns in Figure 2. Also, if we set parameters *α* and *β* to 75000 and 1000, respectively, we can still achieve all the firing patterns of Figure 2, but in order to have more realistic firing patterns, the exact values of *α* and *β* are also added to Table 5.

### B. Phase Portrait

The dynamical analysis of our proposed hyperbolic model is done by investigating the phase portrait and *F*-*I* curves based on (1) and (2).  Figure 8(a) shows the phase portrait of the hyperbolic model for a periodic tonic spike. As can be seen, the spiking cycle at the beginning moves slowly towards −62 mV, which is the reset potential. This is due to the prespike reset time and the dynamics of the recovery variable *H*. When the upstroke of the spike starts, the spiking cycle line shows a linear trajectory towards the maximum peak. Once the spiking cycle line reaches the *H* nullcline at 30 mV, it will reset to the starting point, causing a periodic spiking behavior. Based on parameter selection, the phase portrait might vary for different spiking patterns. In Figure 8(b), the *F*-*I* curve or the changes in the frequency of firings based on the input current are depicted. The *F*-*I* curve was obtained for the tonic spike pattern. The reset voltage of the neuron was set to −62 mv, which is higher than the resetting voltage of the Izhikevich model and AdEx model (−65 mV and −70 mV, resp.). Because of the lower reset voltage of hyperbolic neurons, the upstroke will start sooner, causing more spikes (higher frequency). For the small values of input current, the neuron will fire at low frequencies, while at 14 *μ*A the frequency of firing increases almost exponentially, which is consistent with previous models [9]. The *F*-*I* curve for the Izhikevich model converges to hyperbolic model as we increase the input current. For the AdEx model, there is very low frequency firing for input current below 20 *μ*A. Increasing the input current in the AdEx model generates a linear relation with frequency of firing and, for input currents more than 70 *μ*A, the frequency of firing reaches its threshold and remains almost constant for higher input currents. The threshold of firing frequency for the hyperbolic model and Izhikevich model happens at 75 *μ*A and 80 *μ*A, respectively.
